# Silencing of rhomboid domain containing 1 to inhibit the metastasis of human breast cancer cells *in vitro*

**DOI:** 10.22038/IJBMS.2018.29788.7191

**Published:** 2018-11

**Authors:** Chunjun Huang, Xiaochun Ji, Yinyin Peng, Minghua Wu, Weizhu Wu, Yong Luo, Gaoxiang Cheng, Ye Zhu

**Affiliations:** 1 Thyroid Breast Surgery, Ningbo Medical Center Lihuili Eastern Hospital, Ningbo, 315000, China; 2 Thyroid Breast Surgery, Ningbo Medical Center Lihuili Hospital, Ningbo, 315000, China

**Keywords:** Breast neoplasms, Lentivirus, Neoplasm metastasis, Rhomboid domain - containing 1, RNA, Small interfering

## Abstract

**Objective(s):**

A growing body of evidence indicates that rhomboid domain containing 1 (RHBDD1) plays an important role in a variety of physiological and pathological processes, including tumorigenesis. We aimed to determine the function of RHBDD1 in breast cancer cells.

**Materials and Methods:**

In this study, we used the Oncomine™ database to determine the expression patterns of RHBDD1 in normal and breast cancer tissues. We performed lentiviral transfection of RHBDD1-specific small interfering RNA into the breast cancer cell lines ZR-75-30 and MDA-MB-231 in order to investigate the effects of RHBDD1 deficiency on breast cancer metastasis.

**Results:**

We found that knockdown of *RHBDD1* inhibited breast cancer cell migration and invasion *in vitro*. Moreover, knockdown of *RHBDD1* promoted epithelial–mesenchymal transition (EMT) by suppressing the expression of MPP2, MPP9, fibronectin 1, vimentin, SRY-box 2, zinc finger E-box binding homeobox 1, and snail family transcriptional repressor 1, and promoting the expression of cadherin 1. Additionally, knockdown of *RHBDD1* inhibited the protein expression and phosphorylation of Akt.

**Conclusion:**

Our data indicate that RHBDD1 overexpression may promote breast cancer metastasis via the regulation of EMT, suggesting that RHBDD1 may be an important regulator of breast cancer metastasis.

## Introduction

Breast cancer is the most common and deadly malignancy among women worldwide. According to the latest data from the United States, an estimated 252,710 new cases of breast cancer were diagnosed (30% of all newly diagnosed cases of cancer in women) and an estimated 40,610 people died of breast cancer (14% of all cancer deaths in women) in 2017 (1). Breast cancer-related morbidity is less common in developing than in developed countries (2). In China, breast cancer accounted for 15% of all newly diagnosed cancer cases (268,600 of 1,779,500 patients) and approximately 7% of all cancer deaths (69,500 of 1,004,400 patients) among females in 2015 (3). Similar to those for patients with other solid tumours, the prognosis and survival rate for patients with metastatic breast cancer are poor (4, 5). Therefore, exploring the potential mechanisms of breast cancer metastasis will be beneficial for the survival and prognosis of patients.

Rhomboid family proteins are highly conserved intramembrane serine proteases that were initially recognized in Drosophila; the proteins were named after the malformed heads caused by their mutation (6, 7). The family members usually contain 6 or 7 transmembrane domains, which are cleaved to generate the active proteins that are involved in a number of biological processes (8). The rhomboid family member rhomboid 1 activates the epidermal growth factor receptor pathway in Drosophila (9). In yeast, rhomboid proteases participate in the remodelling of the mitochondrial membrane (10). In addition, rhomboid proteases may be involved in the proliferation and apoptosis of cancer cells. For instance, rhomboid 5 homolog 1 and rhomboid domain containing 2 are overexpressed in multiple human cancer cells, compared with their expression in healthy tissues, and they are closely associated with the progression of and unfavourable prognosis for breast cancer (11-14). 

Rhomboid domain containing 1 (RHBDD1) was identified by Wang *et al*. in 2008, based on its high expression in human testes and its ability to cleave the pro-apoptotic Bcl-2 family protein Bcl-2 interacting killer (15). Furthermore, RHBDD1 is highly expressed in human cancers, such as colorectal cancer, glioblastoma, hepatocellular carcinoma, and chronic myeloid leukaemia; silencing *RHBDD1* inhibits the proliferation of these tumour cells (16-19). However, the function of* RHBDD1* in breast cancer is unknown. In this study, we used lentiviral vectors to deliver *RHBDD1*-silencing small interfering RNAs (siRNAs) to the breast cancer cell lines ZR-75-30 and MDA-MB-231. We investigated the influence of *RHBDD1* silencing on breast cancer cell metastasis and invasion.

## Materials and Methods

***Analysis of the Oncomine™ database***

We analysed *RHBDD1* expression in breast cancer in five datasets – Ma Breast 4, Karnoub Breast, Finak Breast, Curtis Breast, and The Cancer Genome Atlas Breast – in the Oncomine™ database (https://www.oncomine.org), as previously described (20).

**Figure 1:**
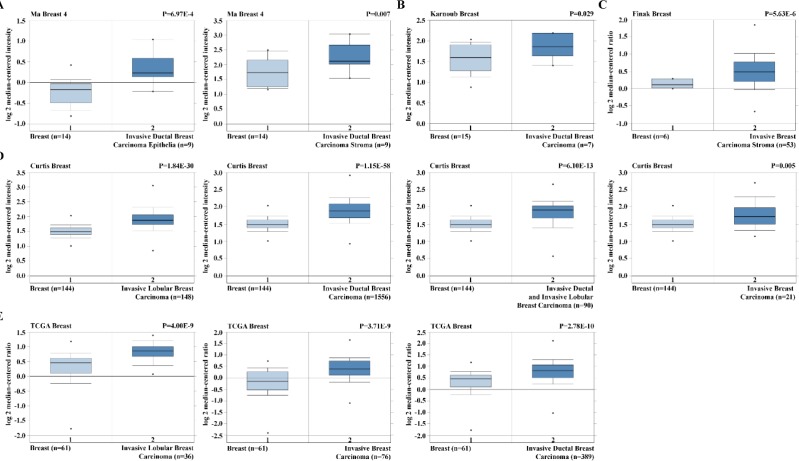
*RHBDD1* expression in breast cancer

We assessed the differences in the expression of *RHBDD1* in invasive breast cancer tissues and in normal breast tissues in the (A) Ma Breast 4, (B) Karnoub Breast, (C) Finak Breast, (D) Curtis Breast, and (E) The Cancer Genome Atlas Breast datasets in the Oncomine™ database

**Figure 2:**
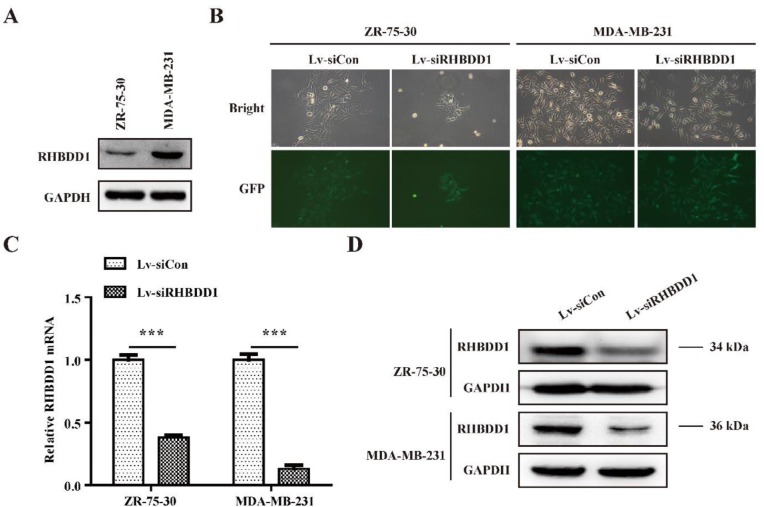
Lentiviral delivery of siRNA silenced *RHBDD1* in breast cancer cells

(A) We measured RHBDD1 protein expression in 2 breast cancer cell lines, ZR-75-30 and MDA-MB-231, by Western blot assay. (B) Representative fluorescence images of ZR-75-30 and MDA-MB-231 cells transfected with Lv-siCon or Lv-siRHBDD1. (C, D) Knockdown efficiency of RHBDD1 in ZR-75-30 and MDA-MB-231 cells, as assessed by quantitative reverse transcription PCR and Western blot assays. ***P<0.001

**Figure 3:**
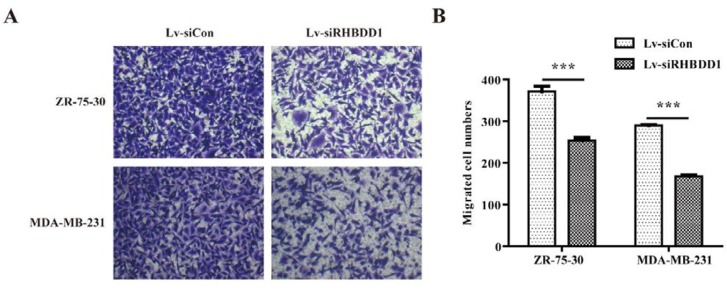
*RHBDD1* knockdown suppressed the migration of breast cancer cells

(A) Representative images from transwell migration assays with transfected ZR-75-30 and MDA-MB-231 cells. (B) Statistical analysis of the number of migrated ZR-75-30 and MDA-MB-231, based on 5 random images. ***P<0.001

**Figure 4:**
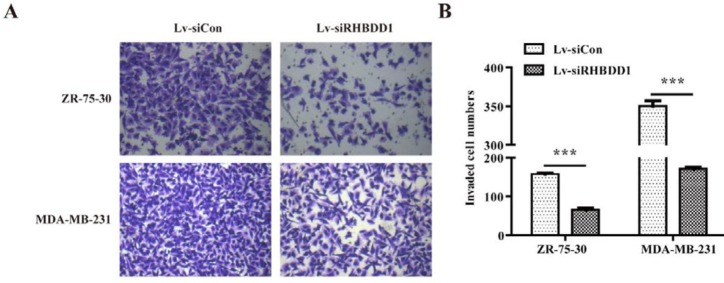
Silencing of RHBDD1 blocked breast cancer cell invasion

(A) Representative images of transwell matrigel assays with ZR-75-30 and MDA-MB-231 cells. (B) Statistical analysis of the number of invading ZR-75-30 and MDA-MB-231 cells, based on 5 random images. ***P<0.001

**Figure 5:**
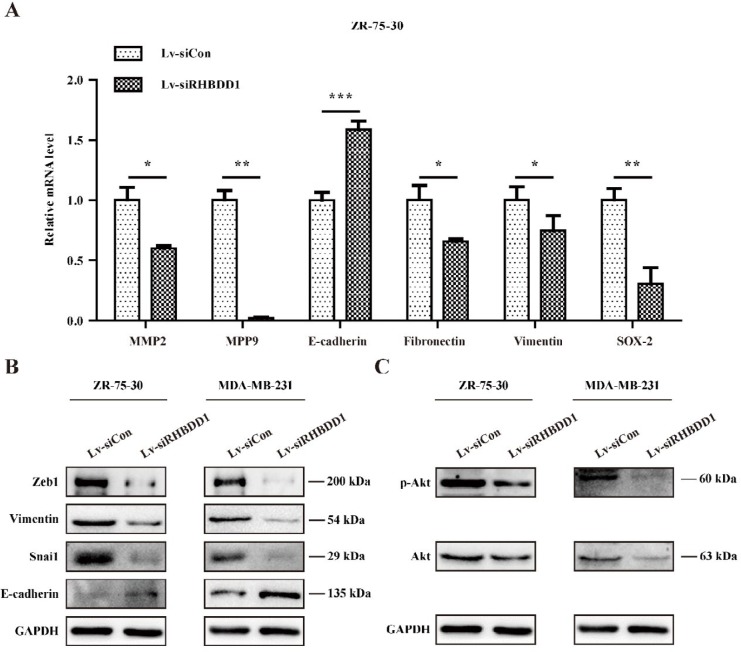
*RHBDD1* knockdown induced EMT of breast cancer cells by inhibiting Akt expression

(A) We analysed the mRNA expression levels of EMT-related genes in ZR-75-30 cells by quantitative reverse transcription PCR. (B) We analysed the protein expression levels of EMT-related genes in ZR-75-30 and MDA-MB-231 cells by Western blot assay. (C) The expression levels of total and phosphorylated Akt in ZR-75-30 and MDA-MB-231 cells as analysed by Western blot assay. *P<0.05, **P<0.01, ***P<0.001. EMT, epithelial–mesenchymal transition

**Table 1:** Real-time PCR primers

Gene	Sequence (5′ to 3′)	
	Forward	Reverse
*RHBDD1*	ATCTGGCTGGGATTCTTGTTG	GGCTGGCTTGTAATGCTCTC
*MMP2*	GGAAAGCCAGGATCCATTTT	ATGCCGCCTTTAACTGGAG
*MMP9*	TTGGTCCACCTGGTTCAACT	ACGACGTCTTCCAGTACCGA
*CDH1*	GACCGGTGCAATCTTCAAA	TTGACGCCGAGAGCTACAC
*CDH2*	GCTCCCTTAATTCCTCAAGTAGTG	TTCAGTCATCACCTCCACCATAC
*FN1*	AATTGCTAGTTTACCGTTCAGAAG	ATGAAGGAAAGGTGGAGGGAAG
*VIM*	ATTCCACTTTGCGTTCAAGG	CTTCAGAGAGAGGAAGCCGA
*SOX2*	GCCGAGTGGAAACTTTTGTC	GTTCATGTGCGCGTAACTGT
*ACTB*	GTGGACATCCGCAAAGAC	AAAGGGTGTAACGCAACTA

***Cell culture***

We cultured the breast cancer cell line ZR-75-30 (Cell Bank of Type Culture Collection of Chinese Academy of Sciences, Shanghai, China) in RPMI-1640 medium (HyClone™, Logan, UT, USA) supplemented with 10% fetal bovine serum (Lonsera, Shanghai, China). We cultured the breast cancer cell line MDA-MB-231 and HEK-293T cells (Cell Bank of Type Culture Collection of Chinese Academy of Sciences, Shanghai, China) in DMEM (HyClone™, Logan, UT, USA) supplemented with 10% fetal bovine serum. All cells were cultured at 37 ^°^C in a humidified atmosphere with 5% CO_2_. 

***Lentiviral vector construction and transfection***

We purchased the *RHBDD1*-targeting siRNA (siRHBDD1) vector and non-Target siRNA Control vector from Sigma–Aldrich (St. Louis, MO, USA). The vectors were separately co-transfected with pVSVG-I and pCMV R8.92 (Sigma–Aldrich, St. Louis, MO, USA) into HEK-293T cells using Lipofectamine® 2000 (Invitrogen, Carlsbad, CA, USA) according to the manufacturer’s instructions. After 48 hr, we collected the supernatants, which contained the recombinant lentiviral vectors with the *RHBDD1*-silencing sequence (Lv-siRHBDD1) or the control sequence (Lv-siCon), and stored them at −80 ^°^C.

We cultured ZR-75-30 (7×10^4^/well) and MDA-MB-231 (8×10^4^/well) cells in 6-well plates overnight, then transfected them with Lv-siRHBDD1 or Lv-siCon at multiplicities of infection of 45 and 60, respectively. Ninety-six hours post-transfection, we assessed the transfection efficiencies of the vectors in the ZR-75-30 and MDA-MB-231 cells by measuring the expression of green fluorescent protein, encoded by the vectors, under a fluorescence microscope (Olympus, Tokyo, Japan).

***Quantitative reverse transcription polymerase chain reaction***

Total RNA was exteracted from the ZR-75-30 and MDA-MB-231 cells transfected with Lv-siRHBDD1 or Lv-siCon using TRIzol® reagent (Invitrogen, Carlsbad, CA, USA). We reverse-transcribed the RNA into complementary DNA using M-MLV Reverse Transcriptase (Promega, Madison, WI, USA). We performed real-time polymerase chain reaction (PCR) with SYBR™ Green PCR Master Mix (Thermo Fisher Scientific, Waltham, MA, USA), according to the manufacturer’s protocol, on a Bio-Rad CFX96™ Real-Time PCR Detection System (Hercules, CA, USA), as previously described (21). We standardized the relative mRNA levels to the levels of the internal control gene β-actin (*ACTB*). The primers are shown in Table 1. 

***Western blot analysis***

The breast cancer cells were lysed with 2x SDS Sample Buffer (Sangon Biotech, Shanghai, China), and quantified the proteins using a BCA Protein Assay Kit (Beyotime, Shanghai, China). Equal amounts of protein was separated from each sample by 10% sodium dodecyl sulphate-polyacrylamide gel electrophoresis, then electro-transferred the proteins to polyvinylidene difluoride membranes (Bio-Rad, Hercules, CA, USA). and blocked the membranes in 5% sealing solution, then incubated with primary antibodies, including anti-RHBDD1 (1:5,000; Sigma–Aldrich, St. Louis, MO, USA), anti-GAPDH (1:50,000; Proteintech, Chicago, IL, USA), anti-zinc finger E-box binding homeobox (ZEB1; 1:1,000; Cell Signaling Technology, Danvers, MA, USA), anti-vimentin (1:1,000; Proteintech, Chicago, IL, USA), anti-snail family transcriptional repressor 1 (SNAI1; 1:2,000; Cell Signaling Technology, Danvers, MA, USA), anti-E-cadherin (also known as cadherin 1; 1:1,000; Proteintech, Chicago, IL, USA), rabbit anti-Akt (1:1,000; Proteintech, Chicago, IL, USA), and rabbit anti-phospho-Akt (1:1,000; Cell Signaling Technology, Danvers, MA, USA). Then, we incubated with the corresponding secondary antibody (1:5,000; Santa Cruz Biotechnology, Santa Cruz, CA, USA). Finally, we visualized the protein bands of interest using Pierce™ ECL Western Blotting Substrate (Invitrogen, Carlsbad, CA, USA).

***Transwell assay***

For cell migration assays, we placed cell suspensions (ZR-75-30: 5×10^4^ cells and MDA-MB-231: 4×10^4^ cells) in the upper chambers of Corning® Transwell® apparatuses (Corning, NY, USA) and 500 µl complete medium in the lower chambers. For cell invasion assays, we placed the cell suspensions (5×10^4^ cells) in Corning® BioCoat™ Matrigel® Invasion Chambers (Corning, NY, USA) and 500 µl complete medium in the lower chambers. After incubation at 37 ^°^C for 24 hr, we fixed the migrated cells in 4% paraformaldehyde (Sigma–Aldrich, St. Louis, MO, USA), then stained in crystal violet (Beyotime, Shanghai, China). Finally, we counted the numbers of migrated cells under a light microscope (Olympus, Tokyo, Japan).

***Statistical analysis***

We used GraphPad Prism version 5.0 (La Jolla, CA, USA) for the statistical analyses. We compared the differences between groups using Student’s *t*-test. The data are presented as the mean ± standard deviation. Differences with *P*<0.05 were considered statistically significant.

## Results

***RHBDD1*** ***was overexpressed in breast cancer cells***

To assess the role of RHBDD1 in breast cancer, we first analysed 5 independent microarray datasets in the Oncomine™ database (22-25). Searches of the datasets showed that *RHBDD1* expression was significantly higher in various types of invasive breast cancers, including invasive ductal breast carcinoma, invasive breast carcinoma, invasive lobular breast carcinoma, and invasive ductal and lobular breast carcinoma, than in normal breast tissues (*P*<0.05, Figure 1). These data indicate that *RHBDD1* is overexpressed in invasive breast cancers and could be involved in the migration and invasiveness of breast cancer.

***Lentiviral vector-mediated silencing suppressed RHBDD1 expression in breast cancer cells***

The expression of *RHBDD1* in 2 breast cancer cell lines was examined. We found that *RHBDD1* expression was higher in MDA-MB-231 cells than in ZR-75-30 cells (Figure 2A). We then transfected ZR-75-30 and MDA-MB-231 cells with Lv-siCon or Lv-siRHBDD1; we confirmed transfection by microscopic analysis of green fluorescent protein expression (Figure 2B). RHBDD1 expression at both the mRNA and protein levels was markedly lower in the ZR-75-30 and MDA-MB-231 cells after transfection with Lv-siRHBDD1 (*P*< 0.001, Figure 2C and D), indicating that we successfully suppressed the expression of RHBDD1 using lentiviral vector-mediated RNA interference.

***RHBDD1 knockdown inhibited breast cancer cell migration and invasion***

The migration and invasiveness of ZR-75-30 and MDA-MB-231 cells transfected with Lv-siCon or Lv-siRHBDD1 were investigated. As shown in Figure 3A and B, fewer Lv-siRHBDD1-transfected than Lv-siCon-transfected cells (ZR-75-30: 254±7 versus 372±13 and MDA-MB-231: 168±3 versus 290±2, respectively; *P*<0.001 for each cell type) migrated in the transwell assay. Similarly, there were fewer invasive cells among the Lv-siRHBDD1-transfected than the Lv-siCon-transfected cells (ZR-75-30: 65±4 versus 158±2 and MDA-MB-231: 171±4 versus 350±7, respectively; *P*<0.001, Figure 4A and B). These results indicate that *RHBDD1* knockdown significantly inhibits the migration and invasiveness of breast cancer cells *in vitro*.

***RHBDD1 knockdown induced epithelial–mesenchymal transition in breast cancer cells***

Given that epithelial–mesenchymal transition (EMT) is involved in cancer metastasis and invasion, the effects of *RHBDD1* silencing on the expression of EMT-related genes were assessed. We found that ZR-75-30 cells in which *RHBDD1* had been knocked down expressed significantly lower levels of *MPP2*, *MPP9*, fibronectin 1 (*FN1*), vimentin (*VIM*), and *SOX2*, but higher levels of E-cadherin (*CDH1*), than the control-transfected cells (*P*<0.05, Figure 5A). In addition, following *RHBDD1* knockdown, both ZR-75-30 and MDA-MB-231 cells expressed lower levels of ZEB1, vimentin, and SNAII proteins, but higher levels of E-cadherin, than the control-transfected cells (Figure 5B). These results indicate that *RHBDD1* knockdown suppresses breast cancer cell metastasis by regulating EMT-related gene expression.

***RHBDD1 silencing suppressed Akt expression and phosphorylation in breast cancer cells***

In order to explore the underlying mechanisms involved in RHBDD1-driven metastasis in breast cancer cells, the effects of *RHBDD1* knockdown on Akt were assessed. We found that knockdown significantly reduced Akt protein expression and phosphorylation in both ZR-75-30 and MDA-MB-231 cells (Figure 5C). These results demonstrate that RHBDD1 regulates breast cancer cell metastasis via the Akt/NF-κB pathway.

## Discussion

Rhomboid proteins have been evolutionarily conserved from early forms of life and they play important biological roles (7). RHBDD1 possesses 4 essential features: the highly conserved rhomboid domain, a cleavage site at the GG motif, susceptibility to the serine protease inhibitor aprotinin, and the GFSGV motif (26). Most studies on RHBDD1 have concentrated on its role in cell proliferation and apoptosis in various cancers (16, 18, 26). However, its function in tumour metastasis, particularly breast cancer metastasis, had gone unreported. Our study provides novel insights into the role of RHBDD1 in breast cancer and, more generally, metastasis.

EMT is associated with the development, metastasis, and invasion of many cancers. During EMT, epithelial cells lose their cell polarity, cell shape plasticity, and adherence to neighbouring cells, and obtain the abilities to migrate and invade tissues (27, 28). In addition, cells lose E-cadherin expression or gain vimentin expression due to the high expression of mesenchymal-related transcription factors, including MPP2, MPP9, SNAI1, and ZEB1 (29, 30). For example, ZEB1 serves as a transcriptional repressor of ‘epithelialness’ by inhibiting the calcium-dependent cell–cell adhesion glycoprotein E-cadherin, thereby triggering EMT (28). We found that the epithelial marker E-cadherin was more highly expressed when *RHBDD1* was silenced, whereas the expression levels of mesenchymal markers (fibronectin and vimentin) and transcription factors (MPP2, MPP9, SNAI1, ZEB1, and SOX2) were lower than in control-transfected cells. 

The Akt/NF-κB signalling pathway plays important roles in the regulation of the migration and invasion of breast cancer (31, 32). The activation of Akt signalling results in cell invasion via EMT (33). In particular, the activation of Akt promotes the release of NF-κB into the nucleus, which induces the activation of transcription factors, such as SNAI1 and ZEB1, and inhibits the production of E-cadherin, thereby enhancing EMT (34-36). We found that *RHBDD1* knockdown suppressed the expression and phosphorylation of Akt. Thus, we speculate that RHBDD1 facilitates breast cancer cell migration and invasion, at least in part, by inducing EMT and regulating the Akt/NF-κB pathway. 

## Conclusion

RHBDD1 may be essential for breast cancer progression. It could be a promising prognostic indicator or therapeutic target for human breast cancer.
